# New developments in the evolution and application of the WHO/IPCS framework on mode of action/species concordance analysis^+^

**DOI:** 10.1002/jat.2949

**Published:** 2013-10-25

**Authors:** M.E. Meek, A. Boobis, I. Cote, V. Dellarco, G. Fotakis, S. Munn, J. Seed, C. Vickers

**Affiliations:** a Chemical Risk Assessment, McLaughlin Centre for Population Health Risk Assessment, 1 Stewart Street, Ottawa, Ontario, Canada K1N 6N5; b Department of Medicine, Imperial College London, Hammersmith Campus, Ducane Road, London W12 0NN, United Kingdom; c National Center for Environmental Assessment, U.S. Environmental Protection Agency, 1200 Pennsylvania Avenue, NW, Washington, DC 20460, USA; d Formerly Office of Pesticide Programs, U.S. Environmental Protection Agency, 1200 Pennsylvania Avenue, NW, Washington, DC 20460, USA; e European Chemicals Agency, Annankatu 18, 00120 Helsinki, Finland; f Systems Toxicology Unit, Institute for Health and Consumer Protection, Joint Research Centre, European Commission, Via E. Fermi, 2749, I-21027 - Ispra (VA), Italy; g Office of Pollution Prevention and Toxics, U.S. Environmental Protection Agency, 1200 Pennsylvania Avenue, NW, Washington, DC 20460, USA; h International Programme on Chemical Safety, World Health Organization, 20 Avenue Appia, CH-1211 Geneva 27, Switzerland

**Keywords:** key events, mode of action, adverse outcome pathway, human relevance framework, modified Bradford Hill considerations, weight of evidence approach, species concordance analysis, cellular response, tissue response, molecular target

## Abstract

The World Health Organization/International Programme on Chemical Safety mode of action/human relevance framework has been updated to reflect the experience acquired in its application and extend its utility to emerging areas in toxicity testing and non-testing methods. The underlying principles have not changed, but the framework’s scope has been extended to enable integration of information at different levels of biological organization and reflect evolving experience in a much broader range of potential applications. Mode of action/species concordance analysis can also inform hypothesis-based data generation and research priorities in support of risk assessment.

The modified framework is incorporated within a roadmap, with feedback loops encouraging continuous refinement of fit-for-purpose testing strategies and risk assessment. Important in this construct is consideration of dose–response relationships and species concordance analysis in weight of evidence. The modified Bradford Hill considerations have been updated and additionally articulated to reflect increasing experience in application for cases where the toxicological outcome of chemical exposure is known.

The modified framework can be used as originally intended, where the toxicological effects of chemical exposure are known, or in hypothesizing effects resulting from chemical exposure, using information on putative key events in established modes of action from appropriate *in vitro* or *in silico* systems and other lines of evidence.

This modified mode of action framework and accompanying roadmap and case examples are expected to contribute to improving transparency in explicitly addressing weight of evidence considerations in mode of action/species concordance analysis based on both conventional data sources and evolving methods.

## Introduction

The mode of action/human relevance framework was developed in initiatives of the International Programme on Chemical Safety (IPCS) of the World Health Organization (WHO) (Boobis et al., 2006, 2008; Sonich-Mullin et al., 2001) and the International Life Sciences Institute Risk Sciences Institute (ILSI-RSI) (Meek et al., 2003; Seed et al., 2005). It derives from earlier work on mode of action in animals by the U.S. Environmental Protection Agency (U.S. EPA, 1996, 2005a) and has involved large numbers of scientists internationally.

Previous development of the mode of action/human relevance framework is described in the publications mentioned above and summarized more recently in Meek and Klaunig (2010). The framework has been illustrated by an increasing number of case studies (more than 30 currently) demonstrating the value of mode of action in evaluating human relevance and life stage susceptibility and guiding dose–response assessment. Documented examples are presented in Table 1. The contribution of the framework has been recognized by the Society of Toxicology, and the framework has been adopted by several international and national organizations and agencies to increase transparency in the assessment of weight of evidence and identification of critical data needs (Meek, 2008, 2009; Meek et al., 2008).

The framework continues to evolve as experience increases in its application to consider systematically the weight of evidence from traditional and evolving methods for assessing toxicity. This includes explicit consideration of the comparative weight of evidence and associated uncertainties for several options for hypothesized modes of action early and throughout the analysis. The critical relevance of the kinetic and dynamic information considered in the mode of action analysis for subsequent characterization of dose–response relationships for effects considered relevant to humans (Boobis et al., 2009; Julien et al., 2009), including choice of chemical-specific adjustment factors (Boobis et al., 2008), has also been amplified. Experience in mode of action analysis has also been instructive in contextualizing appropriate application of information from evolving methods of toxicity testing at different levels of biological organization as a basis for more efficient testing strategies.

## Objectives

This paper has been prepared as an addendum to the previous WHO/IPCS guidance on mode of action/human relevance analysis (Boobis et al., 2006, 2008). While the underlying principles and methodology are similar, the guidance has been updated to reflect recent developments. Some of these developments result from advances in toxicity testing and non-testing methods, and some reflect evolving experience in mode of action/species concordance analysis (additionally referred to herein as mode of action analysis). More detailed information on the nature of systematic hypothesis generation and weight of evidence considerations in mode of action analysis with illustrative case examples is included in the earlier publications referenced in Table 1.

This paper also expands the scope of previous manuscripts to reflect increased understanding of the role of mode of action/species concordance analysis in integrating information from different levels of biological organization. In addition, while early focus of mode of action analysis related to increasing transparency in documenting an operative mode of action with a reasonably high degree of confidence as a basis for risk assessment and regulatory decision-making, the current paper addresses a much broader range of contexts. These include implications for priority setting and testing strategies for both individual chemicals and chemical categories where a less refined analysis and/or higher uncertainty may be acceptable. Summaries of cases selected to illustrate examples of broad application in a research/regulatory context are included here. Readers are referred to the cited documentation for more detailed information on the data analysis for these cases.

Both cancer and non-cancer effects are addressed, in recognition that their separation in earlier publications reflected principally evolving experience in mode of action/human relevance analysis rather than variation in conceptual premise. In fact, mode of action analysis facilitates harmonization of cancer and non-cancer assessment. Harmonization in this context refers to a biologically consistent approach to risk assessment for all endpoints, for which exploration of biological linkages is critical to ensuring maximal utility of relevant information. Often, for example, cytotoxicity in an organ is a critical key event that may lead to an increase in cell proliferation and tumors at the same site.

## Background/Terminology

Mode of action, as previously defined, is a biologically plausible series of key events leading to an effect (Sonich-Mullin et al., 2001). Originally, mode of action was considered principally in the context of late-stage key cellular, biochemical and tissue events. A key event is an empirically observable step or its marker, which is a necessary element of the mode of action critical to the outcome (i.e., necessary, but not necessarily sufficient in its own right); key events are measurable and reproducible. The mode of action framework is based, then, on the premise that any human health effect caused by exposure to an exogenous substance can be described by a series of causally linked biochemical or biological key events that result in a pathological or other disease outcome. (The term mode of action implies no judgment about adversity of effect, though for risk assessment application, the relevant identified or presumed effects are most often considered adverse.) While originally and often simply conceptualized and illustrated as a linear series of key events, in reality, mode of action involves interdependent networks of events with feedback loops. Disease outcomes are initiated or modified within these networks. Differences in networks between and within human and animal populations account, in part, for interspecies differences and human variability.

Early key events in hypothesized modes of action are most often related to chemical characteristics—i.e., those characteristics of structure and/or physicochemical properties that promote interaction of the substance with biological targets. Later key events are less chemical specific and more often an expected consequence of progression of earlier key events (e.g., regenerative proliferation resulting from cytotoxicity).

An adverse outcome pathway is conceptually similar to a mode of action. It was initially described by the computational ecotoxicology community (Ankley et al., 2010) and has been adopted within an international initiative to document, develop and assess the completeness of potentially predictive tools for adverse ecological and human health effects (OECD, 2012). A focus of adverse outcome pathways is on the initial associated chemically mediated “molecular initiating event,” equivalent to an early key event in a mode of action.

**Figure 1:**
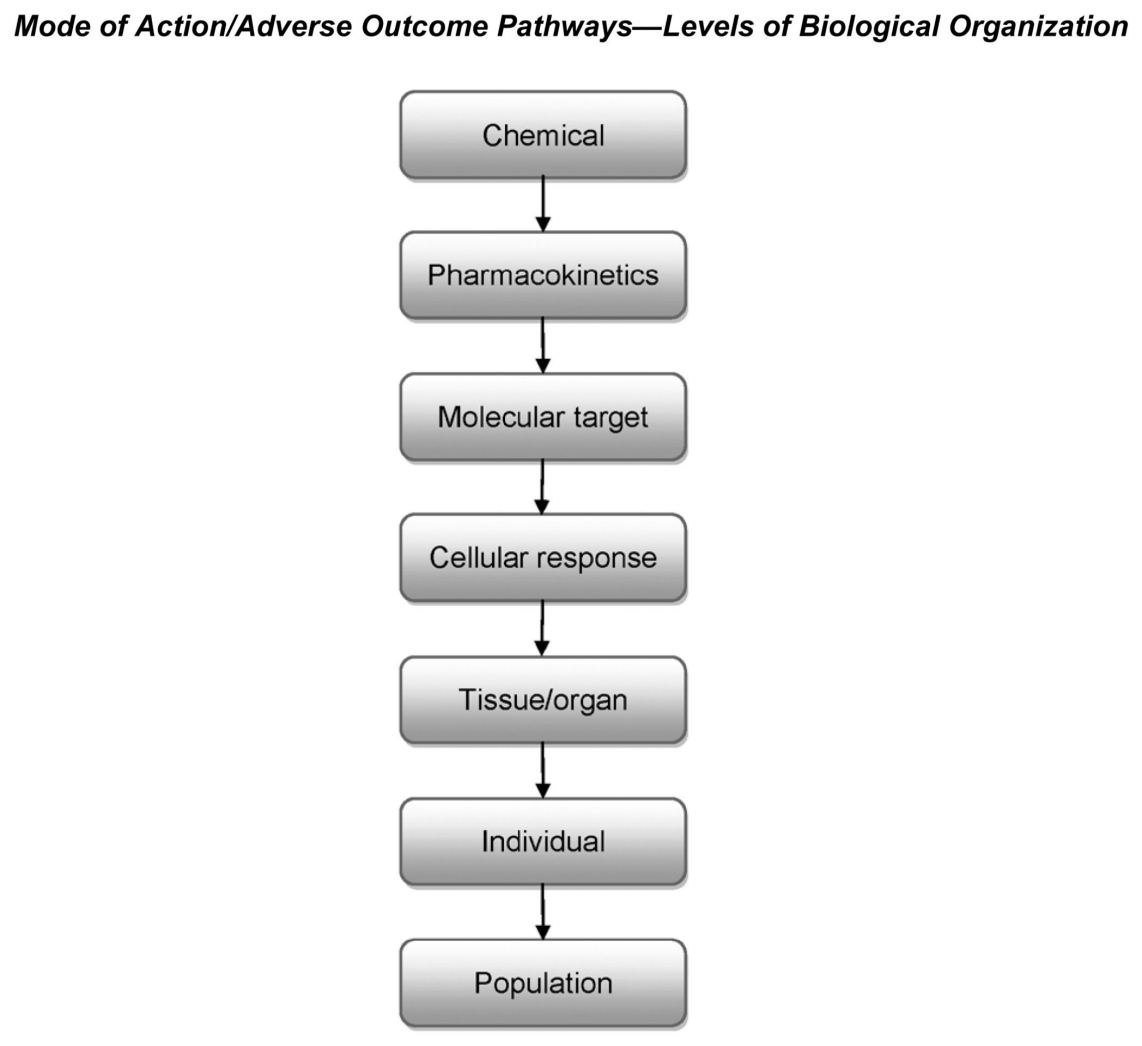
Different levels of biological organization in mode of action analysis. Confidence in an hypothesized mode of action generally increases with increasing evidence at higher levels of biological organization.

The terms mode of action and adverse outcome pathway should be interchangeable, representing essentially the subdivision of the pathway between exposure and effect in either individuals or populations into a series of hypothesized key events at different levels of biological organization (e.g., molecular, subcellular, cellular, tissue) (Fig. 1). (The term toxicity pathway, introduced by the U.S. National Research Council in 2007 [NRC, 2007], essentially focuses on a subset of early events leading to an effect at the molecular and cellular levels. These events can be considered critical upstream elements of a more expansive mode of action description of how a chemical can affect human health.) The distinction between mode of action and adverse outcome pathway is artificial, a result principally of experience in the human health versus ecological communities, though it has sometimes been stated incorrectly that, unlike adverse outcome pathway, mode of action does not extend from the individual to the population level. It should be noted, though, that the term mode of action, *per se*, does not imply adversity of outcome. Mode of action, as defined here, could apply equally well to effects that are not adverse, such as therapeutic interventions or health benefits (e.g., from nutritional supplements). Also, focus on human health risk assessment has traditionally been on (often later) key events that provide quantitative information relevant to intraspecies and interspecies extrapolation and life stage susceptibility for dose–response analysis, compared with the molecular initiating event in ecological health assessment. For this reason, considerations relevant to weight of evidence analysis may differ.

Appropriately, given their conceptual similarity, it has been proposed that the weight of evidence for both hypothesized modes of action and adverse outcome pathways should draw upon modified Bradford Hill considerations (Hill, 1965). This proposal was based on a desire to increase transparency and consistency in organizing, linking and integrating information at different levels of biological organization into a more efficient, hypothesis-driven approach to chemical data generation and assessment and use of non-test (e.g. read-across and grouping of chemicals) and *in vitro* methods.

However, there are a number of limitations that remain to be addressed in the proposed reliance on modified Bradford Hill considerations for documentation of mode of action where focus has been on the molecular initiating event (i.e., structure–activity modeling). For example, weight of evidence for hypothesized modes of action in human health risk assessment has traditionally relied heavily on the modified Bradford Hill considerations of concordance of dose–response relationships between key and end events. In addition, influential in mode of action analysis is specificity, which in this context has related to experimental verification that a key event is causal. And while experience in mode of action analyses for documented (adverse) effects in human health risk assessment can inform consideration of weight of evidence for hypothesized modes of action or adverse outcome pathways, based on early key or molecular initiating events, to date, information on dose–response concordance and specificity has not been available in characterizing weight of evidence for hypothesized adverse outcome pathways. This detracts considerably from transparency in documentation of their supporting evidence.

## Mode of Action Roadmap

There is growing recognition of the need for more efficient methods and strategies to assess the hazards, exposures and risks of the wide array of chemicals to which humans are exposed. This has been reflected in, among others, progressive regulatory mandates in Canada, the European Union and, more recently, the Asian Pacific region to systematically consider priorities for risk management from among all existing chemicals (see, for example, Council of Labor Affairs, Taiwan, 2012; Dellarco et al., 2010; European Commission, 2006; Hughes et al., 2009; Lowell Center for Sustainable Production, 2012; Meek and Armstrong, 2007). This necessitates focus on efficiently prioritized chemicals and endpoints, rather than the traditional time- and resource-intensive series of standard *in vivo* toxicology studies. It also requires the development and integration of information on key events within (hypothesized) modes of action very early in the evaluation process that will enable effective use of data collected from lower levels of biological organization and non-test methods, such as (quantitative) structure–activity relationships ((Q)SAR) and read-across *in vitro* assays.

**Figure 2:**
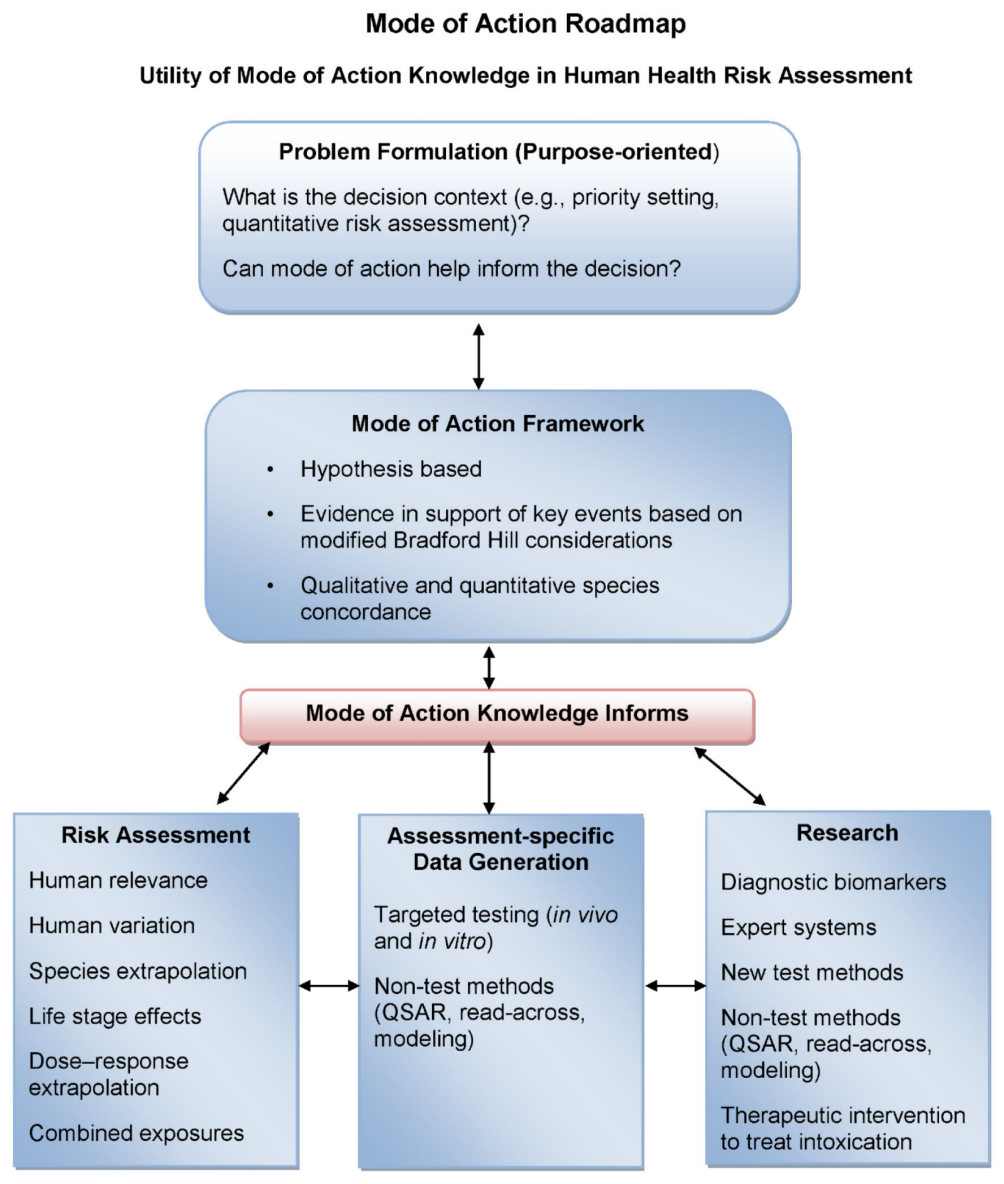
Mode of action roadmap illustrating the use of mode of action knowledge in human health risk assessment. The extent of analysis is tailored to the issue under consideration through iterative analysis and consultation among the assessment, management and research communities.

Figure 2 presents a “mode of action roadmap” to illustrate the iterative process whereby principles and concepts of mode of action analysis can be applied throughout human health risk assessment, with the extent of the analysis being tailored to the issue under consideration. Critical to this more tailored consideration of appropriate testing and assessment strategies is formal, transparent consultation with risk managers, with public accountability, where possible, for the relevant extent of resource investment to address the problem at hand (i.e., problem formulation).

**Figure 3:**
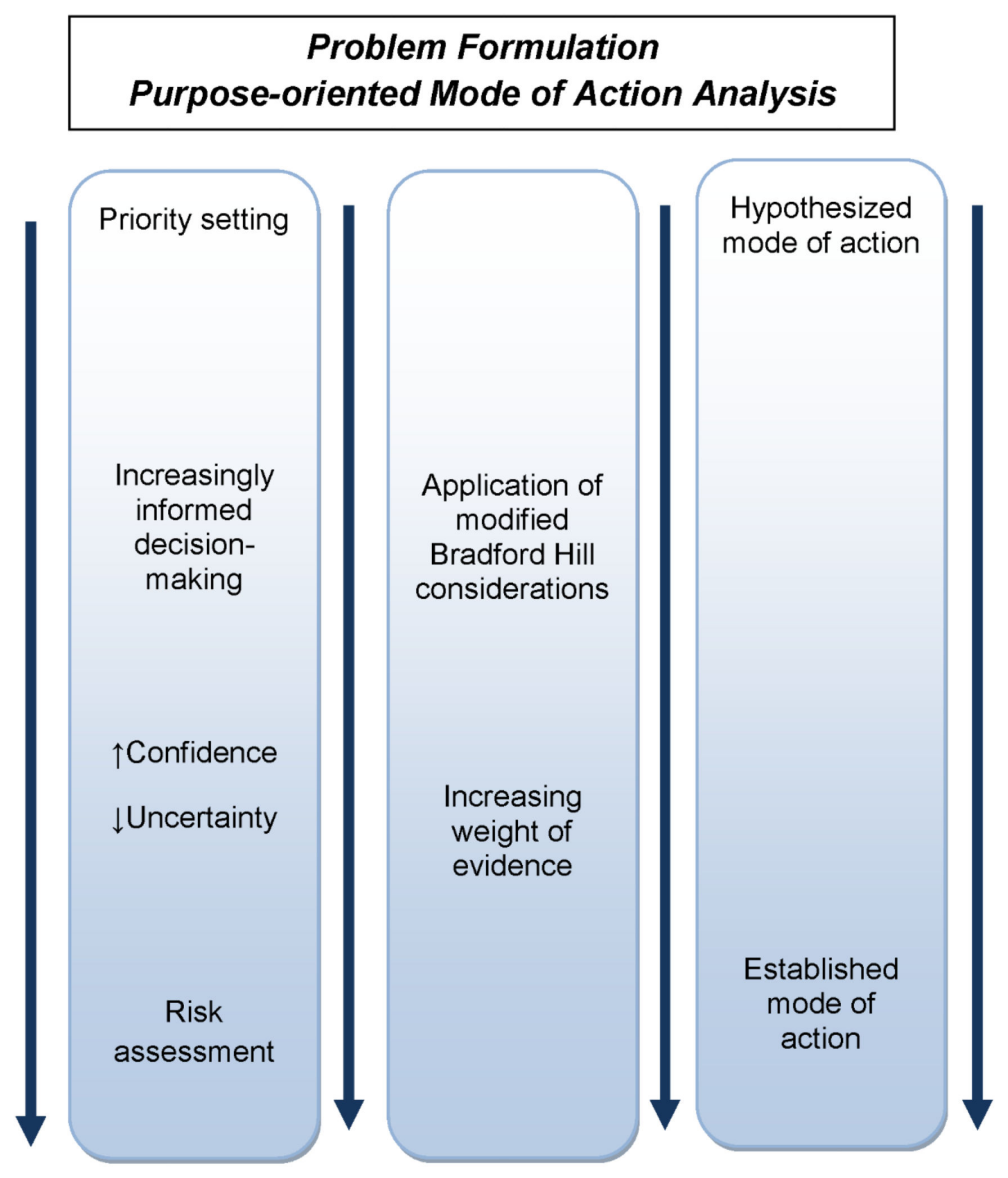
Confidence/uncertainty in “fit for purpose” mode of action/species concordance analysis: correlation of confidence/uncertainty with extent of weight of evidence.

Problem formulation (Fig. 3), the first step in the roadmap (Fig. 2), involves consideration of the risk management scope and goals in relation to relevant exposure scenarios, available resources, urgency of the assessment and the level of uncertainty that is acceptable. This includes consideration of appropriate methods and endpoints for hazard assessment and a mode of action analysis plan tailored to the nature of the decision to be made. For example, decisions concerning chemical prioritization for testing and/or assessment will likely allow for higher levels of uncertainty than those related to establishing regulatory standards. In problem formulation, then, the complexity of the envisaged mode of action analysis is tailored to the context of decision-making; approaches are necessarily flexible and iterative, permitting efficient identification and generation of the essential information to serve as a basis to assess and manage risks appropriately.

The second step in the roadmap (Fig. 2) is to assimilate and consider, in iterative fashion, information on mode of action in the “Modified framework” (see below). This entails hypothesis-based analysis of the weight of evidence for operative key events based on the modified Bradford Hill considerations and qualitative and quantitative concordance of the key events within and between species (Boobis et al., 2006, 2008; Meek et al., 2003; Seed et al., 2005). Early consideration of hypothesis-based key events in the mode of action during problem formulation facilitates incorporation of data from different sources and provides a framework by which it can be organized, integrated and linked at different levels of biological organization (Fig. 3). This includes information generated by evolving methods, such as those targeting cell signaling pathways. The amount of detail and “linearity” characterizing the key events within a hypothesized mode of action can vary as a function of the toxicity of interest, existing knowledge and risk assessment or testing needs.

The mode of action analysis, completed to address the goals outlined during problem formulation, informs one or more of three analytical domains (shown at the bottom of Fig. 2):

1. risk assessment, including qualitative and quantitative human relevance and variability (e.g., effects at various life stages and within susceptible subgroups), dose–response extrapolation and potential for combined effects of chemicals;
2. hypothesis-based targeted testing or application of non-test methods to meet the objectives specified in problem formulation, including efficient grouping of chemicals and consideration of read-across, (Q)SAR modeling or appropriate testing within a category approach to fill data needs; and
3. research priorities relevant to the development of new test and non-test methods, biomarkers and expert systems that feed back to the risk assessment and therapeutic intervention strategies (for intoxication).

As depicted in the roadmap (Fig. 2), mode of action analysis is envisioned as an iterative hypothesis generating and testing process that defines how to assess or test strategically based on risk management needs. As analyses are completed, the problem formulation, testing strategy and risk assessment can be further refined for the decision context.

This iterative process can be illustrated with the following hypothetical example for which there are considerable data on hazard. While this example draws on a relatively extensive data set, it provides a model for considering significantly fewer data on similar compounds, if they are taken into account from the outset in problem formulation. Initially, a risk manager requests that a risk assessment for the general population be conducted for chemical X, for which exposures of potential concern are those through drinking water. In relatively extensive (traditional) toxicity studies (including a cancer bioassay), chemical X has caused liver tumors in rodents. There is controversy regarding the relevance of this particular tumor type for human health risk assessment, and, based on the preliminary mode of action/species concordance analysis in problem formulation, the risk manager is informed that knowledge of the mode of action of induction of tumors in the relevant dose range could inform conclusions on human relevance. Conduct of appropriate studies to address important data needs and uncertainties in the mode of action analysis can then be considered collectively by the risk manager/risk assessor in a refined problem formulation, depending on resources available and time frame for completion.

If additional generation of data is deemed appropriate, the assessment enters the “research” portion of the roadmap, but with a focused effort on generating data relevant to the mode of action/risk assessment question at hand. The targeted relevant mechanistic data that would inform additional assessment and/or management do not require full knowledge of the mechanism, but rather often, quantitative information on determinants of key events, as a basis to predict interspecies differences and human variability better. Upon completion of relevant studies and subsequent mode of action/species concordance analysis, the risk manager is informed of the conclusion (i.e., whether data are considered sufficient to support the hypothesis that the tumors are unlikely to be of relevance to humans).

A potential variant includes the scenario that since the initial problem formulation, the risk manager has become aware that several other related chemicals co-occur with the substance of interest, which may be appropriate for consideration in the same category with chemical X in the risk assessment. The risk manager is informed that the rationale for inclusion of other category members would be strengthened if the same mode of action was suspected; relative potency could then be considered through targeted testing of an early key event. The assessment process now enters the “assessment-specific data generation” portion of the roadmap. Problem formulation can be an iterative process; thus, the results of the targeted testing would further inform the risk manager as to which chemicals within the category are hypothesized to act via the same mode of action, and therefore which should be included for read-across in a combined risk assessment. The assessment process then enters the final “risk assessment” portion of the roadmap.

## Modified Framework

The mode of action framework addresses two key questions. The first is whether there are sufficient data to hypothesize, with an acceptable level of confidence, a mode of action for a known or suspected toxicological outcome. The second is the extent to which such a mode of action would, or is likely to, operate in humans at relevant exposure levels (species concordance analysis).

The framework can also be used in two quite different ways, the first reflecting how it was initially developed, for relatively data-rich chemicals. In this case, causal key events related to an observed (adverse) effect associated with a specific chemical exposure are identified as a basis to utilize available data on kinetics and dynamics maximally to inform relevance to humans and subsequent dose–response analysis; this is referenced below as “Application of the mode of action framework for observed (adverse) effects” and reflects historical experience as is illustrated in many of the case studies currently available. Following problem formulation (Figs. 2 and 3), then, a decision may be taken that a mode of action analysis would be of value in addressing an observed toxicological response for which the margin between measures of hazard and estimated human exposure is such that it warrants additional refinement of the assessment.

The second way in which the framework can be applied is based on information on key events from appropriate *in vitro* and *in silico* systems to predict and assess potential modes of action and potential consequent (adverse) effects (referenced below as “Application of the mode of action framework in hypothesizing (adverse) effects”). The outcome of such an analysis may be the development of a plausible case to predict an (adverse) effect based on knowledge of putative key events or, alternatively, the probable exclusion of certain (adverse) effects, based on an absence of a likelihood of perturbation leading to relevant key events.

In this context, mode of action comprises a series of causally associated key events leading to, potentially leading to or hypothesized to lead to an (adverse) effect. Hence, there can be only one mode of action for one chemical or group of chemicals leading to a specified effect under a given set of conditions. However, different chemicals, or the same chemical under different conditions (e.g., at higher doses or concentrations), may produce the same effect via different modes of action. An example would be the generation of site of contact tumors in the nasal cavity. One chemical may produce such an effect through cytotoxicity and subsequent cell replication promoting spontaneous mutations, another through DNA reactivity leading to gene mutations promoted by regenerative proliferation secondary to cytotoxicity, and a third through interaction with DNA leading to early mutations. In addition, early key events in competing pathways may, or often, converge to produce the same late key event (and outcome). Each mode of action comprising a series of key events for a given response will be different, but some of the key events may be common to other modes of action leading to the same response. The nature of the key events involved will have an impact on the shape of the dose–response curve and on interspecies and intraspecies differences.

**Figure 4:**
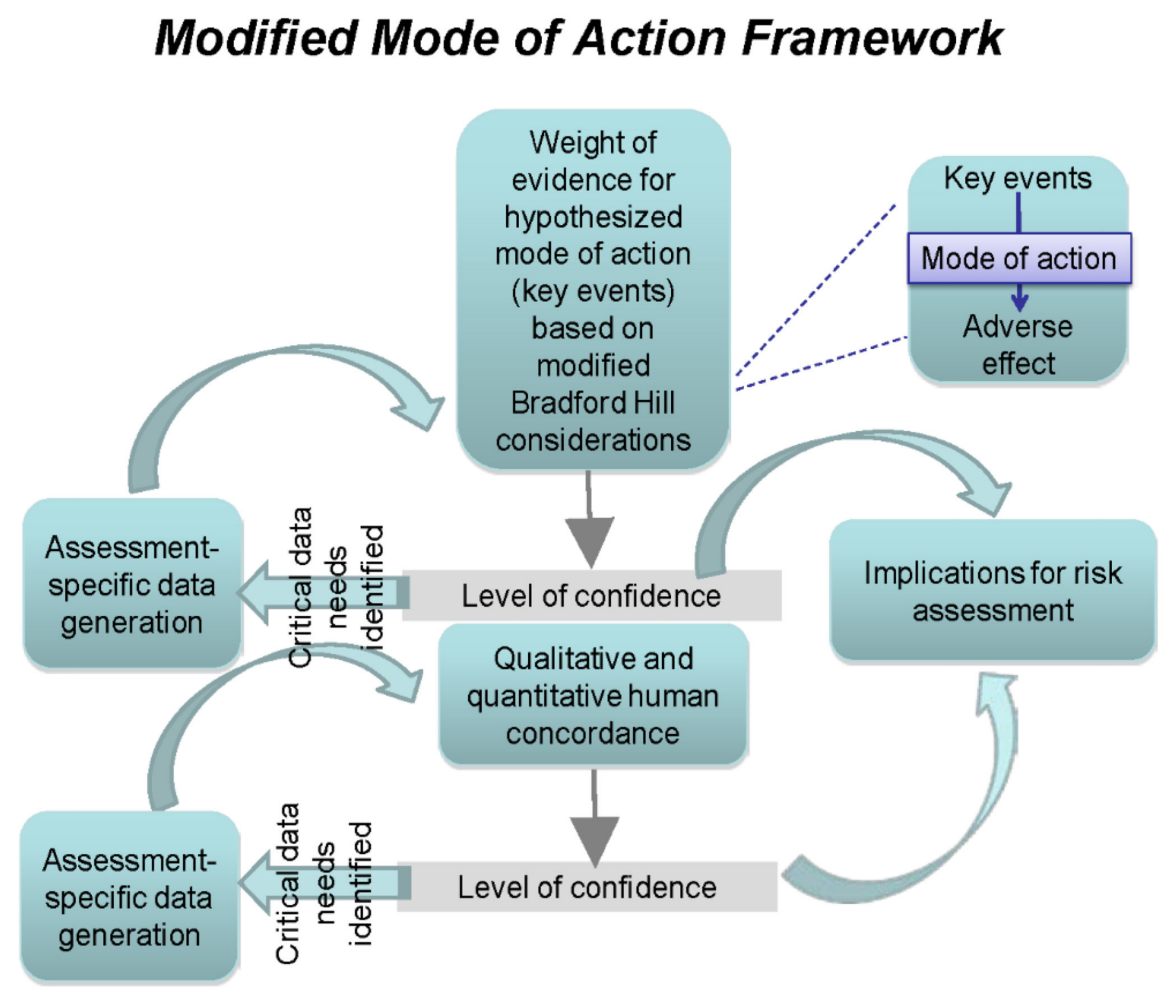
Modified mode of action/human relevance framework and its relation to data needs identified and risk assessment. The application of the framework to assess for observed (adverse) effects and in hypothesizing (adverse) effects is illustrated. The iterative nature of the analysis and the importance of expressing uncertainty are also highlighted.

The modified mode of action framework is outlined in Fig. 4 and explained in further detail below.

### Application of the Mode of Action Framework for Observed (Adverse) Effects

Only this first approach was addressed in the previous descriptions of the WHO/IPCS/ILSI-RSI mode of action/human relevance framework (Boobis et al., 2006, 2008; Meek et al., 2003; Seed et al., 2005), from which further detailed information can be obtained. Extension of the approach through application to help construct more predictive groupings of chemicals was subsequently highlighted in Carmichael et al. (2011). A key aspect of the approach, as illustrated through case studies, is that there should be an unequivocal effect to address before embarking on a mode of action analysis. Hence, problem formulation will have identified the (critical) effect(s) of concern to be considered in the analysis.

In general, mode of action analysis applies to a single effect in a single tissue. In essence, there is one mode of action leading to an effect of interest in the relevant organ for a given substance. This mode of action entails several key events, each of which may result from different, (sometimes) competing mechanisms and/or pathways, although these converge at a late stage to produce the (adverse) effect. It is important, then, to robustly synthesize available information based on multidisciplinary input in hypothesizing potential modes of action. In addition, in the absence of information to the contrary, site concordance between animals and humans is generally assumed, at least as an initial premise. This is often the case, for example, for many non-genotoxic carcinogens that act through perturbation of physiological processes. Similarly, for many non-cancer endpoints, site concordance between test species and humans is a reasonable first assumption, based on considerations of biological plausibility and chemical-specific mechanistic data.

However, there are exceptions to this general principle. Consistent with species- and tissue-specific variation in metabolic activation and detoxification, site concordance for DNA-reactive carcinogens or other effects for which metabolism is critical is often poor. Similarly, for some non-cancer effects induced through a pleiotropic response, such as those that are endocrine mediated, site concordance should not be assumed, but rather considered, based on available mechanistic data and knowledge related to biological plausibility.

These possibilities would need to be scoped at the outset of any mode of action analysis. In such cases, it may be that mode of action analysis would benefit from considering multiple sites in the same evaluation. However, care must be taken to ensure that the mode of action for each effect is likely to be the same, which will not always be the case.

**Figure 5:**
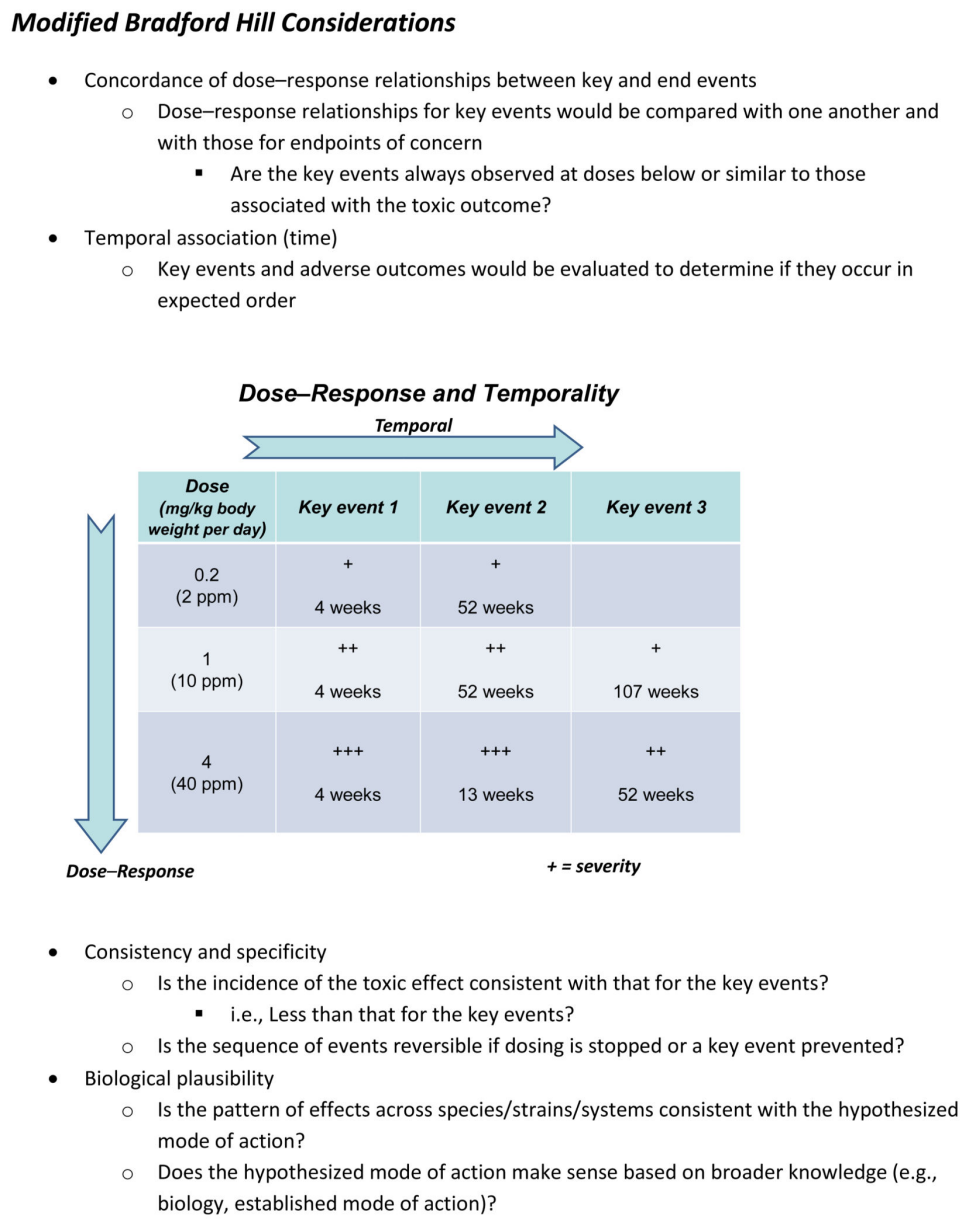
An illustration of the modified Bradford Hill considerations for weight of evidence of hypothesized modes of action. The illustration represents evolution of these considerations based on increasing experience in application in case studies and training initiatives internationally. Specific questions being addressed by each of the considerations are offered as a basis potentially to increase common understanding and consistency in their application in mode of action analysis.

**Figure 6:**
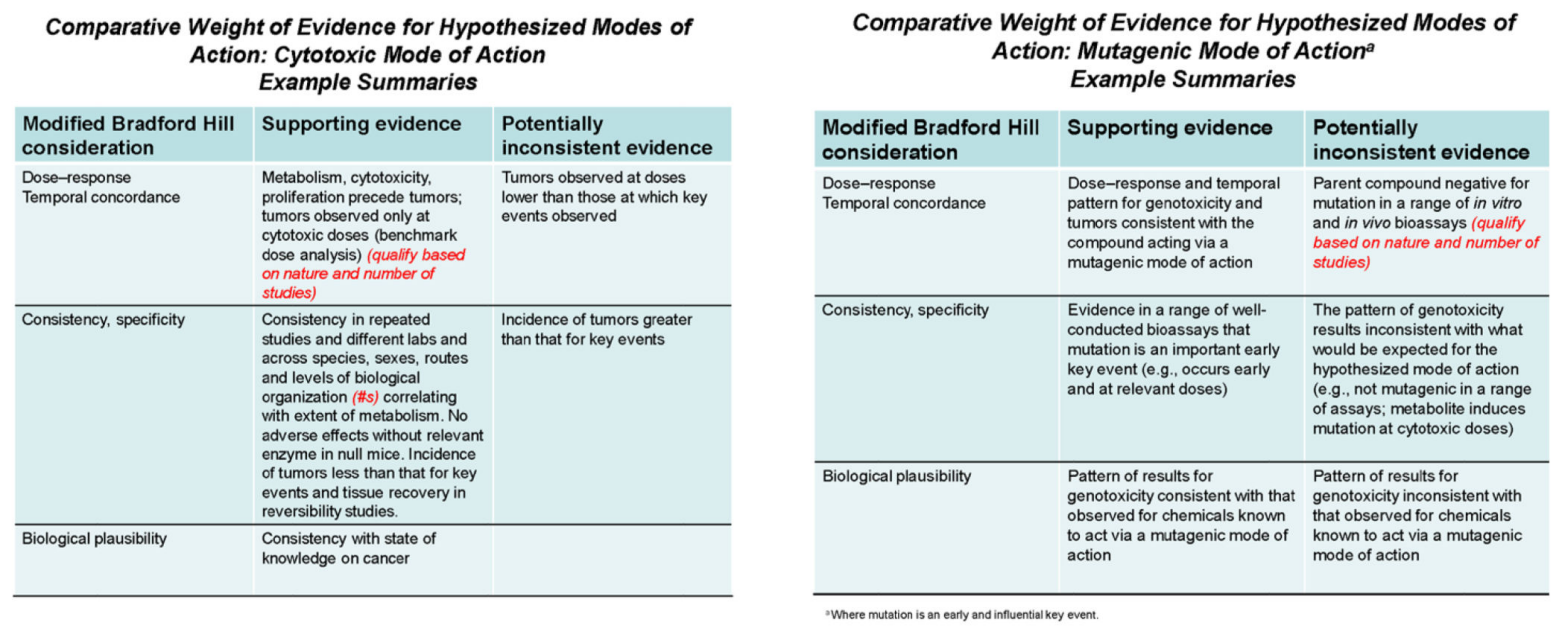
An example of comparative weight of evidence for hypothesized cytotoxic and mutagenic modes of action. Information in each of the columns provides an overview of the extent and nature of the available data and its cohesiveness. Particularly important in interpretation of relative weight of evidence is the nature and extent of data that may be inconsistent with an hypothesized mode of action. In this particular case, the extent of inconsistent data is considerably less for a hypothesized mode of action where mutation is likely to be secondary to cytotoxicity than for a mutagenic mode of action (i.e., where mutation is an early and influential key event). Indeed, the pattern of data on genotoxicity is completely consistent with a cytotoxic mode of action. This would lead to the conclusion that there is greater confidence in the chemical acting by a cytotoxic than by a mutagenic mode of action.

**Figure 7:**
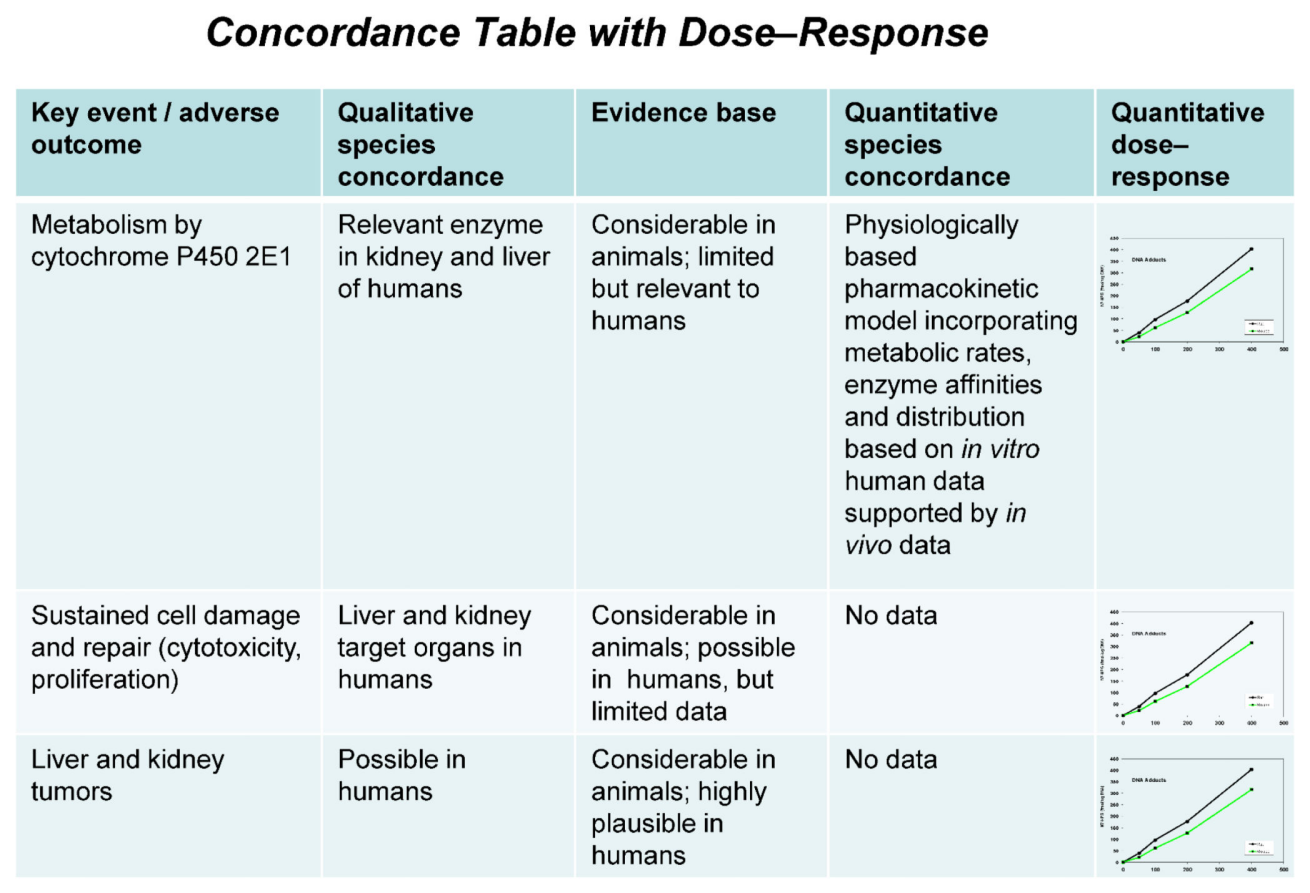
An illustration of a concordance table including dose–response curve. The kinetic and dynamic data considered in assessment of mode of action are directly relevant to dose–response analysis, which takes into consideration dose–response relationships for each of the key events.

Mode of action analysis relies upon biological plausibility and coherence. The weight of evidence for a hypothesized mode of action is addressed based on the Bradford Hill considerations, proposed originally to examine causality of associations observed in epidemiological studies, but later modified in WHO/IPCS and ILSI-RSI publications on the mode of action/human relevance framework (Boobis et al., 2006, 2008; Meek et al., 2003; Seed et al., 2005) and additionally evolved, here. The original templates for consideration of the weight of evidence for a hypothesized mode of action were based on consideration of traditional measures of toxicity, such as biochemical and histopathological parameters in experimental animals. These templates have been adapted here (Figs. 5–7) to reflect additional experience gained in the application of the framework in an appreciable number of case studies over the past decade and as a basis potentially to encompass additional early key events from evolving methods to reliably predict human health outcomes. Based on this experience, robust consideration of dose–response relationships and temporal concordance for early key events will be important in documenting weight of evidence for proposed adverse outcome pathways.

Relevant considerations include dose–response relationships and temporal concordance between specified key events and outcome, consistency (of, for example, the incidence of key events and outcome and changes in causally associated key events), specificity (in the context of essentiality of key events and reversibility) and biological plausibility, based on coherence with the state of knowledge.

In relation to dose–response relationships and temporal concordance, a key event cannot play a role in an (adverse) effect if it is manifest only after toxicity has occurred or if it occurs only at doses higher than those inducing toxicity. The same applies to late key events relative to early key events. There is often a close relationship between dose and time dependency, so that the higher the dose, the earlier a key event is observably affected, and vice versa. This pattern of dose–response and time–response relationships can be invaluable in assessing weight of evidence for a hypothesized mode of action and its key events or how different key events are interrelated. Systematic consideration of dose–response relationships and temporal concordance between key events and (adverse) effects, as illustrated in Figure 5, encourages early assimilation of relevant information from the broader database of both short- and long-term studies, or from different non-animal test systems, in a mode of action context.

More detailed discussion on all of the modified Bradford Hill considerations when applied in the mode of action analysis for observed (adverse) effects is provided in the previous publications on the mode of action/human relevance framework and will not be repeated here. Application and weighting of these considerations continue to evolve as a basis to additionally increase consistency and transparency in assessing weight of evidence in mode of action/species concordance analysis.

It is essential at the outset of mode of action/species concordance analysis that all reasonably plausible modes of action be considered. These include those modes of action that have previously been associated with the relevant effect and any series of key events that logically presents because of available experimental information. The case for each plausible mode of action should be evaluated systematically from the outset, using the modified Bradford Hill considerations.

Weight of evidence for alternative hypotheses should be considered and assessed comparatively. Figure 6 illustrates such an evaluation. Based on relative weight of evidence, it can be determined whether one mode of action could be considered with reasonable certainty to explain the (adverse) effect. Where it is not possible to exclude one or more modes of action, critical data needs could be identified as a basis to inform relevant research that could reduce uncertainty concerning the causal key events within a mode of action, depending on the needs and urgency of the assessment as considered in problem formulation.

The degree of confidence in the outcome should be specified, and each step in the mode of action analysis should be accompanied by a list of the critical uncertainties (i.e., lack of knowledge) and associated data needs, prioritized on the basis of their likely impact, if filled, on weight of evidence and implications for subsequent dose–response analysis.

The comparative analysis of weight of evidence for hypothesized modes of action based on the modified Bradford Hill considerations is followed by statements on the likelihood of each being operative to induce the critical effect. Alternatively, depending on the needs and urgency of the assessment addressed in problem formulation, plausible modes of action should be considered as a basis to contrast strengths and weaknesses of different approaches to quantification of interspecies and intraspecies extrapolation in dose–response modeling. This enables risk managers to distinguish best supported options (i.e., those that are most certain), which is critical in increasing transparency in separating science judgment (i.e., considerations based on experienced consideration of the relevant science base) from science policy determinations (e.g., embedded conservatism in human health risk assessment, incorporated to increase public health protection). Characterization of this nature also contributes to consistency across weight of evidence considerations in different mode of action analyses.

An important objective of framework analysis, then, is the description of the critical sources of uncertainty and characterization of their impact on conclusions concerning weight of evidence for various hypothesized modes of action and their relevance to humans, as a basis particularly for identification of priorities for generation of more or better data. Sensitivity of the estimate to various assumptions can also be tested, and/or available quantitative data relevant to key uncertainties can be analyzed.

Following mode of action analysis and consideration of the associated uncertainties, several outcomes are possible, as illustrated in Figure 4. There may be sufficient information to conclude that a hypothesized mode of action is supported by the available evidence to explain the effect of concern and that the key events for this mode of action have been clearly identified. Where there is insufficient information to reach a conclusion with adequate confidence that a hypothesized mode of action explains the (adverse) effect of concern, appropriate research to address identified critical data needs should provide suitable information to enable confirmation or otherwise of the hypothesized mode of action, through iterative application of the framework. Finally, it may be that at the conclusion of the analysis a hypothesized mode of action is rejected and no other mode of action logically presents itself. In such instances, it may be necessary to proceed with the risk assessment empirically, using relevant information that has been obtained during the analysis of the mode of action—for example, dose–response and time–response information on the endpoint itself, or relevant kinetic and dynamic data.

An important objective of mode of action analysis is to identify those key events that are likely to be most influential in determining potential qualitative and quantitative differences within and between species—that is, key events that are dose and rate limiting. This is addressed in species concordance analysis and is illustrated in Figure 7. Where it has been possible to conclude that a hypothesized mode of action is adequately supported by the available information with an acceptable level of confidence, it is necessary to consider the extent to which such a mode of action would, or is likely to, operate in humans. Species concordance analysis starts with a statement on the level of confidence in the weight of evidence for the hypothesized mode of action under consideration and associated uncertainties. The extent of this analysis is necessarily dependent upon the test system(s) in which key events have been measured, being less for those that best represent humans.

Consideration of mode of action also enables identification of early events or indicators of susceptibility that could be measured in humans (i.e., biomarkers); for example, if there is sufficient information to support early key events such as metabolic activation to a reactive metabolite, this directs attention to the relevant parameters in humans, as a basis to predict interspecies (based on comparison of the relevant parameters between humans and animals, scaled as appropriate) and intraspecies differences (based on consideration of the relevant parameters within different subgroups of the population). Consideration of potential key events also contributes to identification of any specific subpopulations (e.g., those with genetic predisposition or life stage differences) that may be at increased risk.

Assessment of concordance is accomplished by systematic consideration of the nature of the key events between and within species, taking into account both chemical-specific and more generic information, such as anatomical, physiological and biochemical variations. Concordance is considered both qualitatively and quantitatively (Fig. 7). On rare occasions, it may be possible to conclude that a mode of action identified in studies in animals is not relevant to humans because of profound qualitative differences identified in experimental investigation; for example, the molecular target necessary for a key event is not present in humans, and there is no functional equivalent. An example would be α2u-globulin, which plays a key role in the renal carcinogenicity of D-limonene (see Case example 1) (Meek et al., 2003). Alternatively, and very infrequently, quantitative differences in key events may be so great as to render the mode of action not relevant to humans at any conceivable exposure to the substance.

If the weight of evidence for the hypothesized mode of action is sufficient and its relevance for risk assessment cannot be excluded, the implications for dose–response analysis and population variability are considered in the context of identified kinetic and dynamic data. Figure 7 indicates the relevance of delineation of key events in hypothesized modes of action considered to operate in humans in subsequent dose–response analysis. In fact, there is a dose–response curve for each of the key events, and risk for the human population is best predicted on the basis of those key events (or a combination thereof) that are likely to be most influential in impacting or preventing risk, taking into account potential interspecies and interindividual differences in kinetics and dynamics as considered in the species concordance analysis. Reliance on earlier key events offers the potential to better characterize and/or acquire data on effects at lower doses or concentrations in human tissues or populations, which are more relevant for risk assessment. It also contributes to the development of more relevant and informative data for human life stages and subpopulations. For the example given in Case example 2, these data could be used additionally in quantitative species concordance analysis, with implications for subsequent dose–response analysis, the identification of critical data needs and the contribution of evolving methods—in this case, well-designed genomic studies - see “Application of the mode of action framework in hypothesizing (adverse) effects” below (see also Table 2).

Mode of action analysis also contributes to the interpretation of relatively extensive epidemiological data sets. For example, information on key events in mechanistic studies can contribute to better understanding of expected (not necessarily similar) target organs in humans. This is relevant to the interpretation of negative epidemiological data based on their power to detect the most likely site of damage in humans taking into account mode of action and interspecies differences in key determinants of key events. It also contributes to the selection of appropriate biomarkers of effect in epidemiological studies and to understanding of variations between life stages and subgroups of the human population (see Case example 3).

If there is appreciable uncertainty about the relevance or applicability of a mode of action, but critical data needs can be identified, it may be possible to obtain such information through conduct of appropriate studies. Table 2 includes the concordance analysis for the example included in Case example 2, illustrating principal areas of uncertainty, where generation of additional data might meaningfully inform the risk assessment.

If it is not possible to establish whether a mode of action would, or is likely to, operate in humans with an acceptable level of confidence, but there is a pressing need for risk management decisions because of the urgency or the nature of the problem, knowledge of dose–response relationships and variability across species may still be of value in later stages of the risk assessment.

The conclusions of the concordance analysis should be accompanied by consideration of associated uncertainty and a statement on the level of confidence that a mode of action would, or is likely to, operate in humans.

### Application of the Mode of Action Framework in Hypothesizing (Adverse) Effects

Lessons learned in mode of action/species concordance analysis for identified effects are also relevant to its application where the (adverse) effect is not demonstrated but could potentially be presumed based on measurement of putative early key events in established modes of action, taking into account lines of available evidence.

Thus, hypotheses about the key events that can lead to the observed (adverse) effect of concern are developed. In contrast, one can also develop hypotheses of potential (adverse) effects that may be triggered by observed putative early key events, based on previous generic knowledge on documented modes of action. Both approaches involve an iterative process of hypothesis testing and data generation.

In this approach, the objective is to identify those modes of action that could plausibly arise from the (series of) key events identified, either because of previous knowledge of their involvement in a mode of action (e.g., for related chemicals for which there are more data) or because a plausible case can be made on the basis of existing biological understanding that such (a series of) events or perturbations may reasonably lead to (adverse) outcomes under certain time- and dose-dependent conditions. The methods used for evaluating putative modes of action will be fit for purpose, which will not necessarily involve one-for-one validation against existing *in vivo* methods. Thus, at the outset, consideration of potential key events in the mode of action plays an integral role both in the choice of experimental methods (*in vivo*, *in vitro* or *ex vivo*) and in data interpretation. Based on the understanding of the causal linkage of putative key events (either observed or anticipated), hypotheses of the likely potential effects of exposure to a chemical are developed in mode of action analysis. Thus, the modified Bradford Hill considerations are just as applicable here, but are not yet well tested.

In terms of quantitative dose–response assessment of the key events, a critical factor is extrapolation of the effect levels *in vitro* or predicted *in silico* to target tissue concentration *in vivo*—for example, by using physiologically based toxicokinetic modelling (referenced as quantitative *in vitro* to *in vivo* extrapolation modelling). Thus, a key consideration is target tissue concentration of the toxicologically active moiety. This approach lends itself well to identification of the causative agent (i.e., parent or metabolite) and readily enables qualitative and quantitative information to be obtained on the enzyme reactions involved. It may be possible to discount human relevance of some putative modes of action based on the margin between effect levels *in vitro* and anticipated target tissue concentrations *in vivo*. This may be particularly important in the short term, when there is substantial uncertainty about the significance of weak signals obtained using *in vitro* methods.

As discussed above, confidence in a mode of action postulated on the basis of putative early key events identified using non-animal methods will depend on the weight of evidence linking these key events with a mode of action for an adverse response from previous studies and on the ability to “calibrate” quantitative changes in the key event against a degree of change known to have adverse consequences. An example would be inhibition of an enzyme involved in neurotransmitter synthesis or degradation. The extent to which this enzyme needs to be inhibited to produce adverse consequences may be known from studies *in vivo* and could then be used to calibrate such changes determined *in vitro* or predicted *in silico*. Integral to this would be knowledge of the extent to which adaptive mechanisms operating *in vivo* are functional *in vitro* or included in the *in silico* model systems.

Formal analysis of site concordance for key events may not be necessary in this approach. Similar to the mode of action analysis for observed (adverse) effects, data may have been generated in tissue-specific model systems or may reflect site-specific key events. Prediction of likely site of effect will require additional considerations, such as the uptake and disposition of the chemical and the activity of causal pathways in different tissues and cell types. For example, if toxicity depends in part upon transport into the target cell to reach a critical concentration, the presence of the transporter in different cell types would be a key consideration in assessing potential site specificity. Similarly, if one of the key events involved inhibition of a specific potassium channel, the tissue distribution of this ion channel would be an important factor in assessing site specificity. Eventually, as knowledge of the biology of the causal pathways increases, it may be possible to use a systems approach to predict likely affected tissues.

Critical to interpretation of data obtained using non-animal methods will be the model system in which information on putative early key events was obtained and whether coverage of more than one key event would be expected. Some key events may be assessed individually (e.g., using *in silico* approaches to predict binding affinity to a receptor), whereas others may be assessed in a more integrated system (e.g., cytotoxicity in a metabolically competent cell system). Alternatively, high-content analysis and bioinformatics may be used to identify those pathways affected by a substance.

In the case of a well-established mode of action, the focus is on determining whether the measured key events provide sufficient evidence to accept the plausibility for the (adverse) outcome without necessarily generating *in vivo* data specifically to demonstrate the (adverse) outcome. Where the mode of action has not previously been established, the possibility that a plausible case can be made because of existing biological understanding should be addressed. Failing this, the likely outcome of such an analysis is the generation of a hypothesis for a possible (adverse) effect, which can then be tested *in vivo*. In any event, once a mode of action is established, the key events are known *a priori* and can then be assessed *in vitro* or *in silico*. Thus, by understanding the likelihood of effects (i.e., initiation of a toxicity pathway) at lower levels of biological organization (e.g., from SARs and *in vitro* models), it can be determined if more expensive and time-consuming testing at higher levels of biological organization (i.e., *in vivo*) is needed, contributing to increasing efficiency in hazard testing of chemicals. Viewed from the opposite perspective, certain *in vivo* testing could be eliminated for substances that show no potential to initiate the chain of events comprising the mode of action for an (adverse) outcome at environmentally relevant concentrations. In other words, tailored testing can be developed according to screening outcomes indicating the potential for (adverse) effects (see Case example 4).

Where data are available on only one or a limited number of key events and the link to an (adverse) effect has not been sufficiently demonstrated, the data may still be of value in helping to rank and prioritize chemicals, as a basis for additional testing and/or decision-making based on likely relative hazard (e.g., relative potency in modulating sodium channels, endocrine disrupting substance prioritization) (see Case example 5).

More broadly, consideration of SARs for specific key events known to be involved in the mode of action of representative chemicals with the same structural features would be invaluable in helping to construct chemical categories and would enhance the reliability of read-across (see Case example 6 on pyrethroids and Case example 7 on aniline).

Information on mode of action, or on critical key events, can also be invaluable in helping to construct assessment groups for conducting a risk assessment of combined exposure to multiple chemicals (Meek et al., 2011; see Case example 6).

One conclusion from the application of the mode of action framework to information obtained using non-animal methods could be that the data are sufficiently robust to support an established mode of action with a known causal relationship to an (adverse) outcome. Alternatively, it may be possible to conclude that whereas information on one or more key events is missing, provision of information on this data gap would enable a putative mode of action to be assessed with confidence. Finally, the available data may be such that it is not possible to postulate any mode of action with an acceptable degree of confidence.

Increasing numbers of data warehouses comprising substantial amounts of curated information on interspecies and interindividual variability in parameters relevant to many key events are becoming available. These warehouses cover a wide range of species- and individual-specific information, including, from human demographics, anatomical, physiological, biochemical, clinical chemical and life stage–dependent parameters, genetic, genomic, epigenetic, transcriptomic, proteomic and metabolomic information, phenotypic variation in cellular and physiological functions, and expression levels and activities of enzymes and transporters of xenobiotic disposition. Such information, together with evolving bioinformatics and computational tools, may facilitate quantitative (both deterministic and probabilistic) analyses of variability and more robust uncertainty analyses. These tools may also enable more effective analysis of the frequency with which alterations of key events and pathways are reported in similar studies, within and across animal species, and among humans. Similarly, they may permit more thorough analysis of dose, exposure durations and response relationships in pathways across studies.

It should be noted that the availability of larger quantities of data on early potential key events to inform mode of action analyses might lend itself to probabilistic assessments and more robust uncertainty analyses.

## Discussion and Conclusions

The WHO/IPCS mode of action/human relevance framework has been updated to reflect experience acquired in its application, as well as extending its utility to emerging areas in toxicity testing and non-testing methods. The underlying principles have not changed, but the scope of the framework has been extended to integrate information at different levels of biological organization and to reflect evolving experience in a much broader range of potential applications. These applications are relevant not only to full risk assessment for individual chemicals, but also to evolving methods for priority setting and assessment to meet increasing demands to more efficiently and accurately assess and manage large numbers of substances. They include read-across and assessment of groups of chemicals and combined exposures. The mode of action/species concordance analysis also informs hypothesis-based data generation and research priorities in support of risk assessment, related not only to (adverse) effects but also to therapeutic intervention strategies.

Envisaged broader application is illustrated in an integrative and iterative roadmap to address needs for assessment identified in formal problem formulation, as a basis to tailor the appropriate extent of mode of action/species concordance analysis. The roadmap, problem formulation and framework are iterative in nature, with feedback loops encouraging continuous refinement of fit for purpose testing strategies and risk assessment.

The relationship between mode of action and the more recently defined “adverse outcome pathway” is also clarified: conceptually, the terms are synonymous, with both representing division of the path between exposure and effect into a series of key events (including early molecular initiating events) for both individuals and populations. However, mode of action does not necessarily imply adversity of effect, as is seemingly implied by the descriptor adverse outcome pathway.

Broader application of the modified mode of action framework is considered in two contexts, including one for which it was originally developed, where the toxicological effects of chemical exposure are known (i.e., when, as a result of problem formulation, there is a desire to perform a mode of action/species concordance analysis for an observed toxicological effect). The outcome of mode of action analysis in this application is acceptance or rejection of a hypothesized mode of action or recommendation for additional targeted research. Various case examples included here illustrate the nature of information required to demonstrate lack of human concordance, the implications of kinetic and dynamic data considered in mode of action analysis for subsequent dose–response analysis and for the design of targeted research studies using new methods (e.g., genomic technologies) and the integration of toxicological and epidemiological data.

The modified framework can also be applied in hypothesizing effects resulting from exposure to a chemical—that is, with information on putative key events in established modes of action from appropriate *in vitro* or *in silico* systems and other lines of evidence to predict and assess the likelihood of a potential mode of action and consequent effects. With the increasing amount of data available from evolving technologies, such as high-throughput and high-content screening assays, QSARs and other computational approaches, it is likely that this latter application of the framework will be of increasing value to the risk assessment community. The considerable experience acquired in the application of the framework in addressing documented (adverse) effects has a meaningful implication to inform the more limited knowledge base in these more predictive applications. This is illustrated in various case examples, including the use of mode of action analysis in prioritizing substances for further testing, in guiding development of more efficient testing strategies and in identifying critical data needs and testing strategies in read-across. In this vein, mode of action considerations should inform further development of research strategies and data generation methods, as well as the development of biomarkers.

The modified Bradford Hill considerations incorporated in framework analysis from its inception are considered a critical element to document, transparently and consistently, weight of evidence for hypothesized modes of action. These considerations have been updated and additionally articulated somewhat here to reflect increasing experience in application for cases where the toxicological outcome of chemical exposure is known. Additional work is also under way to further simplify and delineate application of the modified Bradford Hill considerations in mode of action analysis. This includes additional articulation of the modified Bradford Hill considerations for weight of evidence as a basis to contribute to common understanding, rank ordering of their importance as well as provision of examples of what might constitute strong versus weak evidence for each, based on acquired experience in mode of action analysis (Meek ME, Palermo CM, Bachman AM, North CM, Lewis RJ, submitted).

A template for extension of the concordance table in the original framework to dose–response analysis is also included, as is one for comparative consideration of weight of evidence for various modes of action based on the modified Bradford Hill considerations. Clear and transparent documentation of uncertainties at each stage of the mode of action analysis is also emphasized, with the objective of being as quantitative as possible regarding the likelihood of a hypothesized mode of action being operative in humans. Additional work to delineate more specifically the appropriate form and content of uncertainty analysis is strongly recommended, consistent with objectives and content of ongoing initiatives in this area.

Experience in mode of action analyses for documented (adverse) effects in human health risk assessment is informative in consideration of weight of evidence for hypothesized effects (referenced as adverse outcome pathways by OECD, 2012), based on early key or molecular initiating events. Based on this experience, development of proof of concept for application of the modified Bradford Hill considerations in more predictive application is strongly recommended. This is particularly important, in view of their significant reliance on demonstration of the essentiality of key events and concordance of dose–response relationships and temporality between early and late key events, information that is often lacking in the more predictive application that is envisaged. Additional collaboration between the health risk and ecological communities in this context is also recommended as a basis to draw on collective experience to increase common understanding and to develop communication and uptake strategies.

In conclusion, the modified framework and accompanying roadmap and case examples are expected to contribute to improving transparency in explicitly addressing weight of evidence considerations in mode of action and species concordance analyses based on both conventional data sources and evolving methods. The broader application envisaged here emphasizes the importance of interaction among the risk assessment, risk management and research communities, as a basis to transition to consideration of data from different levels of biological organization in fit for purpose mode of action analysis (e.g., prioritization vs. full assessment), while also highlighting the need to anchor data from evolving technologies and research. Development of the modified mode of action framework has also highlighted the conceptually identical mode of action and adverse outcome pathway and the resulting need for the research and environmental and human health risk assessment communities to move forward together to develop rigorous, efficient and transparent methodologies to meet increasingly progressive mandates to test and assess, more efficiently and more effectively, much larger numbers of chemical substances in commerce.

## Figures and Tables

**Table 1:** Case studies illustrating various modes of action and implications for dose–response assessment

Mode of action	Case study	Reference
Tumors of various organs associated with mutagenic modes of action	Ethylene oxide 4-Aminobiphenyl	Meek et al. (2003) Cohen et al. (2006a)
Mammary tumors associated with suppression of luteinizing hormone	Atrazine	Meek et al. (2003)
Thyroid tumors associated with increased clearance of thyroxine	Phenobarbital Thiazopyr	Meek et al. (2003) Dellarco et al. (2006)
Bladder tumors associated with the formation of urinary tract calculi	Melamine	Meek et al. (2003)
Liver/kidney tumors associated with sustained cytotoxicity and regenerative proliferation	Chloroform	Meek et al. (2003)
Acute renal toxicity associated with precipitation of oxalate	Ethylene glycol	Seed et al. (2005)
Androgen receptor antagonism and developmental effects	Vinclozolin	Seed et al. (2005)
Nasal tumors associated with DNA reactivity and cytotoxicity	Formaldehyde	McGregor et al. (2006)

**Table 2:** Concordance analysis of key events in the mode of action associated with induction of bladder tumors in rats by cacodylic acid (Cohen et al., 2006; U.S. EPA, 2005b).

Key event	Qualitative concordance		Quantitative concordance	Confidence/uncertainty
	
	Rats	Humans		
Reduction of cacodylic acid (dimethylarsinic acid, or DMA^V^) to the highly cytotoxic metabolite, dimethylarsinous acid (DMA^III^), in urine	Yes: *In vivo* studies detecting DMA^III^ in urine at concentrations that would produce cytotoxicity after DMA^V^ is administered.	Plausible: Evidence following DMA^V^ exposure too limited to draw conclusions, but DMA^III^ shown to be present following human exposure to inorganic arsenic.	Formation of less DMA^III^ in urine of humans compared with rats. Significant levels of additional metabolite trimethylarsine oxide (TMAO) in rodents; detected in humans only at very high doses of inorganic arsenic. DMA^V^ is a poor substrate for the arsenic(III) methyltransferase (AS3MT) in humans.	Considerable evidence in animals; limited in humans.
Urothelial cytotoxicity	Yes: Scanning electron micrographs of rat urothelium; *in vivo* cytotoxicity findings correlate closely with *in vitro* studies.	Human evidence from *in vitro* studies of urothelial cells, potential to occur *in vivo* in humans if sufficient DMA^III^ is formed.	Variation between humans and rats in transport of DMA^V^ across cell membranes. Similar magnitude of response of human and rat epithelial cells to DMA^III^. Interspecies differences could be taken into account in dose–response analysis through physiologically based pharmacokinetic modeling and use of chemical-specific adjustment factor for dynamics.	Considerable consistent evidence that the metabolite leading to urothelial cytotoxicity is DMA^III^ and that cytotoxicity is a rate-limiting key event; quantitative species differences in key events (mode of action) can be taken into account.^a^
Regenerative urothelial proliferation	Yes: *In vivo* 5-bromo-2'-deoxyuridine labeling index data.	No human evidence, but potential to occur in humans if sufficient cell killing is produced and sustained.		Considerable evidence in animals, although some inconsistencies in the data that can be accounted for by variability across different laboratory studies.
Development of urothelial tumors	Yes: Responses in rats but not mice.	No epidemiological data: Only if humans were exposed to doses of DMA^V^ that are sufficiently high to lead to cytotoxic levels of DMA^III^ in the urine.		Strong and consistent evidence supporting the sequence of key events postulated for the development of rat bladder tumors. Good understanding of species differences impacting key events. Evidence in humans is weak. Mode of action is qualitatively plausible in humans, presuming sufficient DMA^III^ is present in the urine.

a Though the biochemical target for cytotoxicity is not understood, this information is not essential for the mode of action.

## Case example 1 Lack of human concordance

D-Limonene provides an example of a data-rich case example for which the mode of action has been established with confidence in the animal model and extensive data are available to demonstrate that it is not relevant to humans (Meek et al., 2003).

Hypothesized key events in the mode of action for species- and sex-specific kidney tumors in male rats were the formation of a stable intermediate, D-limonene-1,2-epoxide, which binds to a protein, α2u-globulin, which accumulates in the renal proximal tubule cells, leading to nephropathy and cellular proliferation, and subsequently tumors, at this site following chronic exposure. There is strong evidence that female rats, laboratory mice and other strains of rats for which there is no evidence of D-limonene-related renal toxicity or tumors do not synthesize or express α2u-globulin.

Consideration of the relevance to humans of the key events leading to renal tumors in the male rat model identified the expression of either α2u-globulin or a homologous protein in humans as critical. After an exhaustive analysis, no protein capable of binding to D-limonene-1,2-epoxide could be identified from human kidney, and therefore it could be concluded that the mode of action leading to kidney tumors in the male rat was not likely to be operable in humans.

This is a rare example of a distinct qualitative difference between the animal model and humans, allowing the possibility to conclude that a mode of action is not relevant to humans. However, it is quite unusual to be able to demonstrate such a qualitative difference. Rather, in the vast majority of cases, such differences will be quantitative, and likely differences in sensitivity of response between animals and humans identified in the mode of action analysis would be taken into account in the subsequent dose–response analysis.

## Case example 3 Role of mode of action analysis in the evaluation of epidemiological data

This case example illustrates the contribution of mode of action analysis when there is substantial human evidence.

Associations between ambient particulate matter exposures and increased cardiovascular mortality were first observed in epidemiological studies without support from animal bioassays, which led to skepticism concerning causality due to the lack of mechanistic underpinning. Subsequent mode of action studies shed light on key events in cardiovascular injury in humans exposed to particulate matter and elucidated interspecies differences and human variability in dosimetry and sensitivity (U.S. EPA, 2009b).

Particulate matter induces adverse effects on the cardiovascular and cerebrovascular systems, such as thrombosis, plaque rupture, myocardial infarction and stroke, via reactive oxygen species, which appear to trigger systemic inflammation through the action of cytokines and other soluble mediators. In general, systemic inflammation is associated with changes in circulating white blood cells, the acute phase response, pro-coagulation effects, endothelial dysfunction and the development of atherosclerosis. The time course of these responses varies according to the acute or chronic nature of the particulate matter exposure; chronic exposures may also lead to adaptive responses.

## Case example 4 Use of mode of action analysis to guide development of more efficient testing strategies

Concepts of mode of action analysis are also helpful in guiding developments in the replacement of *in vivo* toxicity testing.

Modes of action can be hypothesized based on reference chemicals/pharmaceuticals where the sequence of key events leading to a specific (adverse) effect is known at a sufficient level of detail, as a basis to facilitate identification of the characteristics and requirements of *in vitro* systems and *in silico* models that could predict early and subsequent rate-limiting key events in an integrated manner. Once dose–response relationships between the key events measured *in vitro* and biomarkers of response and ultimately adverse outcome *in vivo* are established for reference chemicals, including the necessary *in vitro* to *in vivo* extrapolation, the toxicity of many other chemicals acting through the same mode of action could in theory be characterized and predicted based on the responses in the *in vitro* systems and *in silico* models.

A large research initiative (“Safety Evaluation Ultimately Replacing Animal Testing,” or SEURAT) is based on this premise (Gocht et al., 2013). The first phase of this program, which is co-funded by the European Commission under its Seventh Framework Programme (FP7) and Cosmetics Europe, spans a 5-year period from 2011 to 2015 and includes six research projects, combining the research efforts of over 70 European universities, public research institutes and companies addressing repeated-dose toxicity in hepatic, cardiac, renal, neuronal, muscle and skin tissues. The strategy involves mode of action analysis to describe how any substance may adversely affect human health and to use this knowledge to develop complementary theoretical, computational and experimental (*in vitro*) models that predict quantitative points of departure for safety and risk assessment.

## Case example 5 Mode of action analysis in prioritizing substances for further testing

There is a great deal of interest in prioritizing chemicals for evaluation of endocrine disruption potential (i.e., how best to focus on those chemicals most likely to cause adverse effects without empirically testing all chemicals of regulatory concern). An expert (QSAR) system was developed to predict estrogen receptor binding affinity using the mode of action (adverse outcome pathway) knowledge (OECD, 2009; Schmieder et al., 2003, 2004; U.S. EPA, 2009a). This pathway is initiated through direct chemical binding to the estrogen receptor, which could plausibly lead to reproductive impairment. The predictive model was developed based on two *in vitro* assays: using a rainbow trout estrogen receptor competitive binding assay to directly measure the chemical-biological interaction and a trout liver slice assay in which the consequences of estrogen receptor activation or inhibition are measurable as a result of tissue uptake and partitioning of the chemical in the presence of xenobiotic metabolism.

## Case example 6 Mode of action in the creation of chemical categories

This example addresses the risk assessment of a new synthetic pyrethroid with the same pesticidal mode of action and insecticidal effects as other members of this structural class of compounds. The critical effect of most pyrethroids is reversible neurotoxicity through interaction with a common target, neuronal sodium channels (reviewed in Soderlund, 2012). This mode of action has been established with confidence, and hence the similarity of the pesticidal mode of action of a new member of this chemical group will provide evidence that the compounds share key events. This can be used to support read-across. The risk assessment of a new pyrethroid could then be based on the assumption that it will share a mode of action with other pyrethroids and its likely relative hazard considered in this manner for a first-tier assessment.

The mode of action involves interaction with neuronal sodium channels (Clark and Symington, 2012; Soderlund, 2012). Hence, interaction with sodium channels is a key event for what is often the critical effect. One could rank existing pyrethroids for their potency in modifying the neuronal sodium channel in a suitably designed *in vitro* system and determine the potency of the new compound in this system (Cao et al,, 2011b; McConnell et al,, 2012). One would also wish to consider basic toxicokinetic aspects, such as absorption (which could be predicted from lipid solubility) (Hou et al,, 2009) and metabolic stability (which could be determined in *in vitro* test systems, such as hepatic microsomal fraction or cultured hepatocytes) (Scollon et al,, 2009). This information could be used, either semiquantitatively or with a physiologically based toxicokinetic model (Knaak et al,, 2012), to inform the choice of reference point from among those of the compounds for which information is already available.

Hence, by using an established mode of action for a structurally well-defined group of compounds with a common toxicophore, it is possible to inform read-across in the early tiers of a risk assessment. This could be refined by evaluating specific key events *in vitro* and using the resulting information to refine the read-across process. In this way, the results of new *in vitro* approaches can be anchored in relevant outcomes by using existing knowledge and concepts.

In addition, such information would help in constructing assessment groups for consideration in the risk assessment of combined exposures to multiple chemicals (Cao et al,, 2011a).

## Case example 7 Use of mode of action analysis to identify critical data needs and testing strategies in read-across

This case example is based on a case study presented at an Organisation for Economic Co-operation and Development (OECD) workshop held in December 2010. It addresses a mode of action related to the formation of methemoglobin and a number of industrial chemicals that are anilines, which vary in the quantity of toxicity data available (European Chemicals Bureau,, 2004). It illustrates how the understanding of the mode of action can focus testing and more effectively fill data needs for data-limited compounds.

Aniline induces methemoglobinemia, which, if severe, can result in hemolytic anemia. Hemolytic anemia is a late consequence of methemoglobinemia and a response to the elimination of circulating red blood cells that contain methemoglobin. Aniline is first metabolized in the liver (probably by cytochrome P450 enzymes) to phenylhydroxylamine. It is further oxidized in red cells, most likely to free radical species, via nitrosobenzene. The iron in hemoglobin is oxidized by the free radical species from Fe^2+^ to Fe^3+^, in which state (i.e., methemoglobin) it cannot bind oxygen. Decreased oxygen results in hypoxia-induced necrosis in tissues that have high oxygen needs. Damaged red blood cells are sequestered by the spleen and are phagocytosed by splenic macrophages, leading to increased red blood cell production by the blood-forming organs, primarily the bone marrow. If the bone marrow cannot keep up with the replacement needs, then extramedullary hematopoiesis occurs as a compensatory response. To determine the potential of the untested anilines to result in hemolytic anemia, *in vitro* testing could be conducted to measure the formation of phenylhydroxylamine and/or methemoglobin.

Thus, the mode of action framework provides a conceptual construct to consider key events at different levels of biological organization plausibly linked to an *in vivo* endpoint of regulatory interest. This allows for the development and use of alternative (*in vitro*) assays to target particular cellular or physiological key events along a specific pathway. Once the mode of action has been established, the key event data can be used for read-across from other chemicals. If a new chemical fits the established mode of action, this existing knowledge can be used to justify a more efficient testing strategy, so not every chemical needs to be evaluated in an *in vivo* test.
